# Attitudes of dental implantologists in Spain to prescribing antibiotics, analgesics and anti-inflammatories in healthy patients

**DOI:** 10.4317/medoral.23103

**Published:** 2019-10-27

**Authors:** Fabio Camacho-Alonso, Daniel Muñoz-Cámara, Mariano Sánchez-Siles

**Affiliations:** 1 DDS, PhD. Department of Oral Surgery, University of Murcia, Murcia, Spain; 2 DDS, PhD. Department of Prosthetic Dentistry, University of Murcia, Spain; 3 DDS, PhD. In private oral surgery and medical practice, Murcia, Spain

## Abstract

**Background:**

The implantologists frequently prescribe antibiotics, analgesics and anti-inflammatories in dental implant surgery. The aims of this study were to evaluate the attitudes of implantologists in Murcia (Spain) to prescribing antibiotics, analgesics and anti-inflammatories in healthy patients during different implant dentistry procedures, and to see how these are influenced by individual dentist’s academic level, professional experience, and ongoing training (attending courses or reading scientific literature on medication use)

**Material and Methods:**

This cross-sectional study included a total of 200 implantologists from the Murcia area (Spain), who each completed a two-page questionnaire consisting of 26 questions.

**Results:**

The implant procedure in which most dentists (n=97) prescribed antibiotics was multiple implant surgery with flap raising, in which 55.6% of these 97 respondents used a prophylactic antibiotic regime for 7 days after implant placement. All subjects (n=200) prescribed analgesics for eight out of the eleven procedures included in the survey and anti-inflammatories in six. Dentists with higher academic levels or longer professional experience prescribed more antibiotics, but those who underwent continuous training (attending courses or reading scientific literature) reduced antibiotic prescription.

**Conclusions:**

Dentists often prescribed antibiotics, analgesics and anti-inflammatories in almost all implant procedures in healthy patients, but ongoing training reduced the frequency of antibiotic prescription in some procedures.

** Key words:**Antibiotics, analgesics, anti-inflammatories, dental implant, oral surgery.

## Introduction

In recent years, the prosthetic rehabilitation of missing teeth by means of dental implants has become a common treatment, which has proved very accepTableto patients, mainly due to its high success rates (1). Althought, several scientific papers (2,3) have affirmed that post-operative infection after dental implant placement is considered an uncommon complication with a prevalence that ranges from 1.6% to 11.5%, and that infection mostly occurs during the first month after implant placement.9 But many oral surgeons apply prophylactic antibiotic regimens that continue for 7 days after implant placement, which may constitute a general tendency to over-prescribe antibiotics in implant dentistry (4). While excessive antibiotic use can have adverse effects such as slight to severe gastrointenstinal disorders, rashes, anaphylaxis, and occasionally even death, one of the major problems with antibiotics is the advance of bacterial resistance to them, now considered a threat to public health. The contribution to antimicrobial resistance made by dental treatment remains unclear but it is estimated that between 7% and 11% of all antibiotics are prescribed by dentists. Many of these are administered following implant-based treatments (5). In implant dentistry, it is not only the possibility of generating antimicrobial resistance through the excessive use of conventional antibiotics that is of concern (beta lactams, clindamycin, macrolides, tetracyclines, and metronidazole, administering regimens that continue for 7 days after implant placement, but also the other regimens proposed in implant-based treatments such as pre-operative prophylactic single or multiple doses, or post-operative single or multiple doses (6). So implant treatments involving antibiotics may constitute a source of increased antimicrobial resistance affecting public health generally. In this context, there is a complete lack of consensus on the use of antibiotics during the different procedures involved: single or multiple implant placement, mucoperiosteal flap raising, immediate placement following dental extraction, sinus lift, bone regeneration techniques, secondary surgery, and prosthetic phases (7).

Meanwhile, concepts regarding the management of post-operative symptoms in implant dentistry have undergone important modification in recent years in response to the advances in our understanding of the physiopathological bases of pain and inflammation, as well the pharmacodynamics of the analgesics and anti-inflammatories used in their treatment. In this way, the tendency is now to prevent post-operative pain and inflammation through pre-operative drug administration, which together with classic post-operative medication will combat post-surgical symptoms effectively (8,9).

As for anti-inflammatory treatment, non-steroidal anti-inflammatory drugs (NSAIDS) are the most widely used drugs in the world, with an estimated average usage of 80 Tablets per person per year (10). For this reason, they have become the second most common cause of adverse reactions to medication after beta-lactam antibiotics (11), with an adverse reaction prevalence of 0.1% to 0.9% among the general population (12). Among the adverse effects of NSAID overuse, the most noTable(due to their frequency, morbidity, and mortality) are gastrointestinal effects (dyspepsia, digestive hemorrhages, and gastroduodenal perforations) (13), renal function disorders, platelet aggregation, and increased cardiovascular risk (14).

In light of these controversial findings and the lack of consensus – only one scientific paper has studied antibiotics prescribing practices in different dental implant procedures (7) – and the fact that there is no published information about implantologists attitudes and practice when it comes to prescribing medication, this study set out to assess dentists’ approaches in Murcia (Spain) to the use of antibiotics, analgesics and anti-inflammatories in healthy patients undergoing different implantological procedures and to analyze how these are influenced by the individual dentist’s academic level, professional experience, and participation in ongoing training through course attendance or through reading scientific literature about drug prescription.

Materials and Methods

- Study design

This transversal observational study included a convenience sample of 200 dentists registered with the College of Dentists of Murcia (Spain), all with at least one year’s clinical experience in dental implant placement. All participants were volunteers and received no remuneration. The study was conducted during the period June 2015 to February 2016.

Inclusion criteria were: dentists registered in Murcia with clinical experience of dental implant placement of at least one year. Exclusion criteria were: subject not a dentist and/or insufficient (less than one year) clinical experience in dental implant placement.

- Sample size calculation

In June 2015, there were a total of 1,060 dental professionals registered with the College of Dentists of Murcia (Spain). Having made contact with these individuals by telephone, or E-mail, a total of 430 subjects were found to fulfill the inclusion criteria. To calculate a representative sample size, a power of 80% was required (5% alpha level), which determined the sample size as 210. After inviting 210 subjects to take part, 10 refused to participate, leaving a final sample size of 200.

- Study questionnaire 

The 200 respondents completed a two-page questionnaire consisting of 26 questions, 115 in face-to-face interviews, 35 by phone, and 50 by e-mail.

The questionnaire comprised eight questions related to experience and training in dental implant placement and 18 related to the use and prescription of antibiotics [based on the survey conducted by Abukaraky *et al*., (7)], analgesics, and anti-inflammatories in healthy patients during different phases of implant-based treatments.

- Statistical analysis

Data were analyzed using the SPSS 20.0 statistics program (SPSS® Inc, Chicago, IL, USA). Descriptive statistics were calculated for each variable. The associations between the different qualitative variables were analyzed using Pearson’s chi-squared test. Statistical significance was accepted for p≤0.05.

## Results

The sample group was made up of 200 dentists (58 men and 142 women) from the Murcia region (Spain), all with experience in dental implant placement of at least one year (mean experience 5.04 ± 3.27 years). Descriptive analysis of the study sample showed that 68% were graduates, 25.5% had completed post-graduate Master’s studies, and 6.5% had graduated as medical doctors. Most of the respondents (77.5%) did not attend ongoing training courses in “the use of antibiotics in implant dentistry,” 81% did not attend (ongoing training) courses in “the use of analgesics in implant dentistry,” and 66.5% did not receive ongoing training in “the use of anti-inflammatories in implant dentistry” (Table 1).

Table 2 shows the different prescription regimens for antibiotics, analgesics and anti-inflammatories administered to healthy patients during different phases of implant-based treatment. The treatment in which the most dentists (n=97) prescribed antibiotics was multiple implant placement with flap raising, in which 55.6% of these 97 subjects used a prophylactic antibiotic regimen for 7 days after implant placement. All the survey respondents (n=200) prescribed analgesics in 8 out of the 11 procedures included in the questionnaire, and two even used them during impression taking. As for anti-inflammatory prescription, all respondents (n=200) prescribed anti-inflammatories in six of the eleven treatments (single implant with and without raising flap, multiple implant with and without raising flaps, direct and indirect sinus lift).

The antibiotic of choice was amoxicillin with clavulanic acid (72.5%) or amoxicillin alone (27.5%). When the patient presented a penicillin allergy, clindamycin was the antibiotic of choice in 100% of prescribing subjects. Paracetamol was the most widely used analgesic (80.5%), while NSAIDS were the most commonly used anti-inflammatories (95.5%) (Table 3).

When the possible influence of academic level, professional experience, and ongoing training (through course attendance and/or reading scientific literature) on the use of antibiotics, analgesics and anti-inflammatories during implant treatment (Table 4), it was found that professionals with post-graduate qualifications prescribed antibiotics more frequently than graduates (with the exception of immediate implant placement in absence of infection, and secondary surgery). Clinical experience of over 10 years influenced antibiotic prescription so that they were more often prescribed in 9 out of the 11 procedures included in the survey. Ongoing training by means of attending courses or reading scientific literature led to less frequent prescription of antibiotics in ten of the 11 procedures investigated.

**Table 1:**
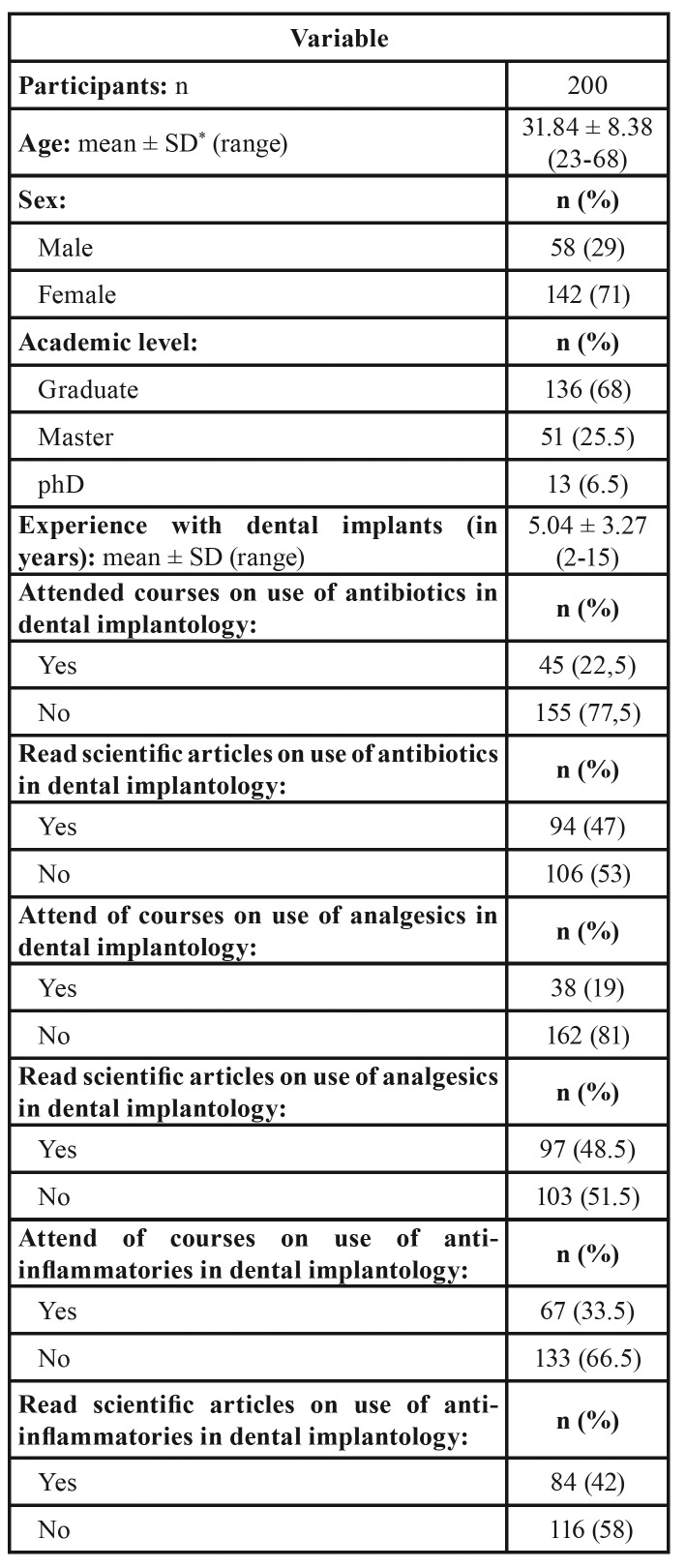
Demographic and professional characteristics of the study sample.

**Table 2:**
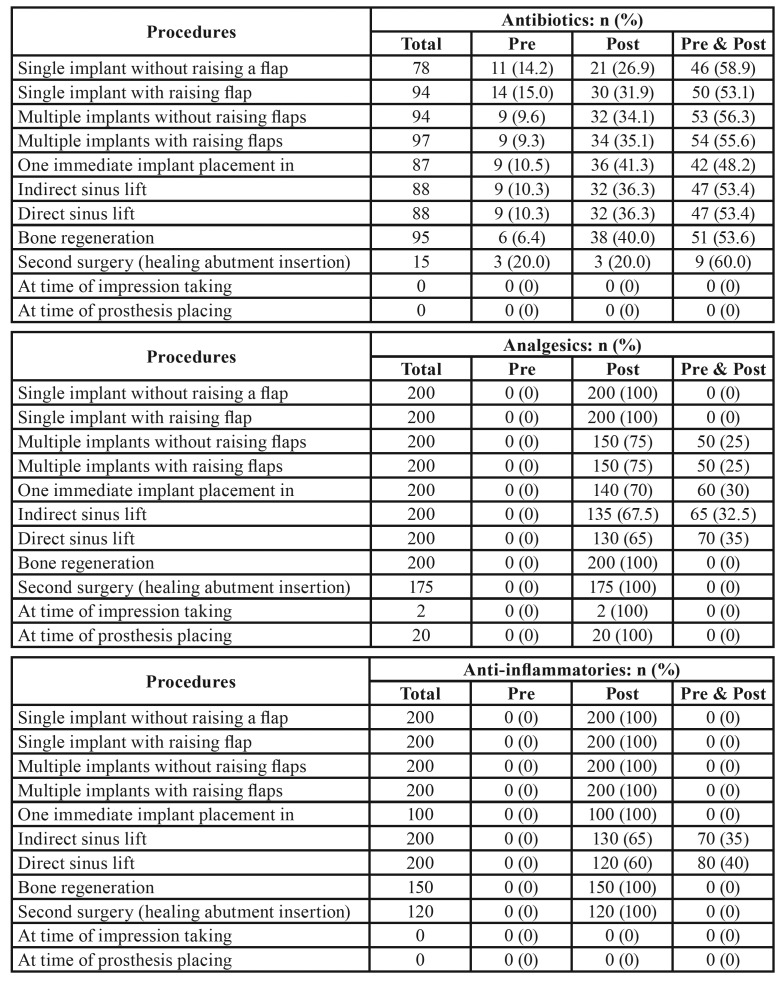
Antibiotics, analgesics and anti-inflammatories prescription choices in different dental implant procedures in healthy patients.

**Table 3:**
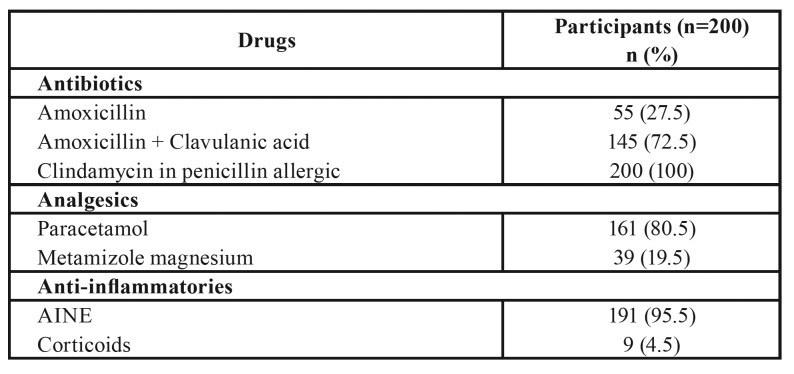
Antibiotics, analgesics and anti-inflammatories prescribed.

**Table 4:**
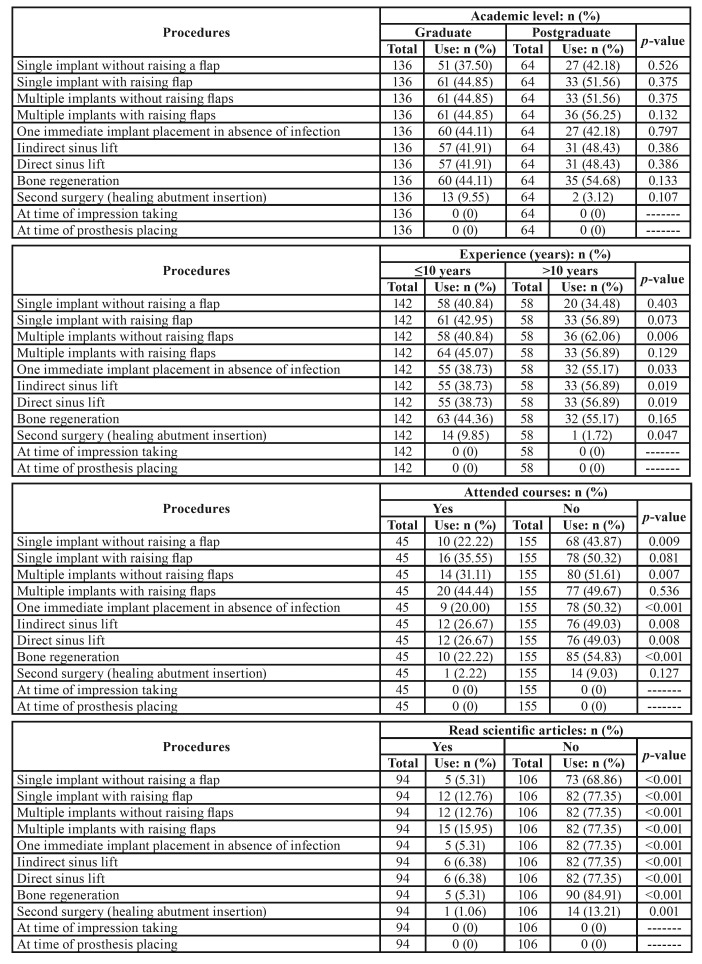
Influence of academic level, experience with dental implants (years), attended courses and read scientific articles on use of antibiotics in different dental implant procedures in healthy patients (Pearson’s chi-squared test).

## Discussion

The exponential growth of implant dentistry has been accompanied by increasing indications for the uses of antibiotics, analgesics and anti-inflammatories in dental implant surgery. Antibiotics are administered to prevent post-operative infection; if an implant becomes infected the likelihood that it will be lost is high (15). So implantologists may consider antibiotic therapy an absolute necessity to prevent infection of the surgical bed by certain types of bacteria (Streptococci, gram-anaerobic and gram-anaerobic bacilli) (16), or even to prevent bacterial proliferation into the bloodstream (6). To attempt to reduce the risk of infection as far as possible during implant placement, diverse regimens of prophylactic systemic antibiotic administration have been recommended, but the use of pre-, post-, or pre- and post-operative antibiotics in dental implant surgery, and their rates of success or failure have hardly been documented in the literature. Very few double-blind case-control studies have been published, mainly for ethical reasons (17).

The present study found that the most widely used antibiotic regimen was the administration of antibiotics pre- and post-operatively in all types of treatment except impression-taking or prosthetic placement (none of the respondents administered antibiotics for prosthetic placement). Comparing the present results with Abukaraky *et al*., (7) the latter found that most Jordanian implantologists performing implant placements only administered antibiotics postoperatively, for single or multiple implants, with or without flap raising. Nevertheless, the study concurred with the present study observing pre- and post-treatment administration for immediate implant placement, indirect or direct sinus lift, or secondary surgery; some of the Jordanian respondents even prescribed antibiotics at the time of impression taking (7%) and prosthetic placement (7.6%).

Assessing the influence of the implantologist’s academic level, professional experience, course attendance or reading scientific articles on medication use, it was observed that dentists with post-graduate qualifications prescribed antibiotics more often than graduates (except for immediate implant placement in the absence of infection, and secondary surgery); similar results were obtained by Wasan *et al*., (18) who found a tendency for dentists with post-graduate training to prescribe more antibiotics than graduates for acute pulpitis, periodontal abscess, dry socket, impacted third molars, and space infection. Regarding the influence of professional experience on antibiotic prescription in implant dentistry, it was found that implantologists with clinical experience of over 10 years prescribed more antibiotics for nine out of the eleven treatments included in the survey, findings which differ from the survey-based transversal study by Bolfoni *et al*., (19) who found that dentists with over ten years experience prescribed fewer antibiotics in patients with endodontic problems (20).

The present study respondents who received ongoing training through course attendance and/or scientific reading prescribed fewer antibiotics in ten of the eleven procedures investigated. Some studies (20) have reported that participation in ongoing training in the form of short courses (lasting a few days) on different dental implant procedures is quite high in Europe, and that the main shortcomings of ongoing training are the lack of courses in bone grafting and implant-supported prosthetics. European implantologists generally call for greater consensus about the content of ongoing training courses (21). As for ongoing training by means of reading scientific articles on the use of antibiotics in implant dentistry, Abukkary *et al*., (7) also observed that the implantologists questioned made good use of literature (79.7%) for ongoing training. But the large quantities of scientific literature on implant dentistry has led authors such as Layton *et al*., (22) to make three recommendations to improve the dissemination of information among dental implant researchers/authors and readers: 1. Authors should improve the quality of their reporting; 2. Journals should allow authors sufficient space in their abstracts to adequately summarize the results, and not impose unrealistic word limits; and 3. Readers should be mindful of these problems when searching for relevant articles and interpreting results.

There is a great deal of controversy around the use of antibiotics in healthy patients undergoing implant-based treatments. Some scientific articles conclude that their use has some effect on the prevention of post-operative complications, and so treatment success rates (23), while others suggest that there are no apparent positive effects deriving from antibiotic therapies (24). To address the current lack of consensus, and determine if antibiotic use, antibiotic prophylaxis, or neither constitutes a correct regimen when placing dental implants in healthy patients, a consensus document was published in 2006 on the use of prophylactic antibiotics in oral surgery. This recommended that the clinician’s criteria should be based on an assessment of risk/benefit, and that the ultimate decision to use antibiotics is a choice that responds to the equation: risk = degree of damage x probability of suffering it (25).

Regarding the use of analgesics and anti-inflammatories, all respondents (n=200) prescribed analgesics for eight out of the eleven procedures included in the questionnaire, and two even used them at the time of impression taking. All respondents (n=200) prescribed anti-inflammatories for six out of the eleven procedures (single implant placement with and without flap raising, multiple implant placement with and without flap raising, direct and indirect sinus lift). The lack of consensus regarding the use of anti-inflammatories and the disparity in pain and swelling management are noTable(26). Trauma to both soft tissues and bone during implant placement necessitates the correct management of pain and post-surgical inflammation and, as with antibiotic use, it is necessary to establish clear guidelines for anti-inflammatory regimens (27).

In our study, the use of phone or e-mail allowed to complete the 200 questionnaires, because 85 implantologists could not complete it by face-to-face inteviews. These technologies improve recruitment for studies about attitudes and behaviors, including increasing professionals contacts, maximizing convenience for participants, and emphasizing interpersonal relationships between researchers and participants (28).

One limitation that the present study suffered was the difficulty of comparing the results with other works, due to the scarcity of similar research into approaches to antibiotic prescription and the complete lack of articles relating to analgesic and anti-inflammatory use. In the future, when a consensus regarding antibiotic, analgesic and anti-inflammatory regimens in implant dentistry procedures has been reached, further studies will be necessary to assess whether implantologists are following the guidelines established.

In conclusion, the present study showed that implantologists frequently prescribe antibiotics, analgesics and anti-inflammatories in different procedures in healthy patients; practitioners with higher academic qualifications and longer professional experience prescribe more antibiotics, while prescriptions are fewer when the implantologist undergoes ongoing training by means of course attendance and reading scientific literature.
